# (2*E*)-3-[3-(Benz­yloxy)phen­yl]-1-(2-hy­droxy­phen­yl)prop-2-en-1-one

**DOI:** 10.1107/S160053681101614X

**Published:** 2011-05-07

**Authors:** Hoong-Kun Fun, Wan-Sin Loh, B. K. Sarojini, V. Musthafa Khaleel, B. Narayana

**Affiliations:** a X-ray Crystallography Unit, School of Physics, Universiti Sains Malaysia, 11800 USM, Penang, Malaysia; b Department of Chemistry, P. A. College of Engineering, Mangalore 574 153, India; c Department of Studies in Chemistry, Mangalore University, Mangalagangotri 574 199, India

## Abstract

In the title compound, C_22_H_18_O_3_, an intra­molecular O—H⋯O hydrogen bond stabilizes the mol­ecular structure, forming an *S*(6) ring motif. The central benzene ring forms a dihedral angle of 64.74 (5)° with the phenyl ring and a dihedral angle of 5.58 (5)° with the terminal benzene ring. In the crystal, mol­ecules are linked into columns along the *a* axis *via* inter­molecular C—H⋯O hydrogen bonds. C—H⋯π inter­actions involving the centroid of the hy­droxy-substituted benzene ring further stabilize the crystal structure.

## Related literature

For the background to chalcones, see: Awad *et al.* (1960 ▶); Coudert *et al.* (1988 ▶); Insuasty *et al.* (1992 ▶, 1997 ▶); Kolos *et al.* (1996 ▶); Samshuddin *et al.* (2010 ▶); Fun *et al.* (2010 ▶); Sarojini *et al.* (2006 ▶); Shettigar *et al.* (2010 ▶); Sharma *et al.* (1997 ▶); Ravishankar *et al.* (2003 ▶, 2005 ▶); Butcher *et al.* (2006 ▶); Narayana *et al.* (2007 ▶); Sarojini *et al.* (2007**a* ▶,b* ▶); Jasinski *et al.* (2011 ▶). For bond-length data, see: Allen *et al.* (1987 ▶). For hydrogen-bond motifs, see: Bernstein *et al.* (1995 ▶). For the stability of the temperature controller used in the data collection, see: Cosier & Glazer (1986 ▶).
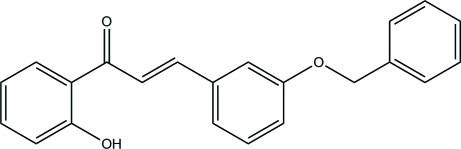


## Experimental

### 

#### Crystal data

C_22_H_18_O_3_
*M* *_r_* = 330.36
Monoclinic, 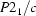

*a* = 6.6343 (1) Å
*b* = 35.7706 (5) Å
*c* = 8.1537 (1) Å
β = 121.879 (1)°
*V* = 1643.12 (4) Å^3^
*Z* = 4
Mo *K*α radiation
μ = 0.09 mm^−1^
*T* = 100 K
0.46 × 0.41 × 0.28 mm

#### Data collection

Bruker SMART APEXII CCD area-detector diffractometer
Absorption correction: multi-scan (*SADABS*; Bruker, 2009 ▶) *T*_min_ = 0.960, *T*_max_ = 0.976
23662 measured reflections
5994 independent reflections
5154 reflections with *I* > 2σ(*I*)
*R*_int_ = 0.024

#### Refinement

*R*[*F* ^2^ > 2σ(*F* ^2^)] = 0.048
*wR*(*F* ^2^) = 0.126
*S* = 1.04
5994 reflections
226 parameters
H-atom parameters constrained
Δρ_max_ = 0.47 e Å^−3^
Δρ_min_ = −0.21 e Å^−3^

### 

Data collection: *APEX2* (Bruker, 2009 ▶); cell refinement: *SAINT* (Bruker, 2009 ▶); data reduction: *SAINT*; program(s) used to solve structure: *SHELXTL* (Sheldrick, 2008 ▶); program(s) used to refine structure: *SHELXTL*; molecular graphics: *SHELXTL*; software used to prepare material for publication: *SHELXTL* and *PLATON* (Spek, 2009 ▶).

## Supplementary Material

Crystal structure: contains datablocks global, I. DOI: 10.1107/S160053681101614X/sj5129sup1.cif

Structure factors: contains datablocks I. DOI: 10.1107/S160053681101614X/sj5129Isup2.hkl

Supplementary material file. DOI: 10.1107/S160053681101614X/sj5129Isup3.cml

Additional supplementary materials:  crystallographic information; 3D view; checkCIF report

## Figures and Tables

**Table 1:** Hydrogen-bond geometry (Å, °) *Cg* is the centroid of the C17–C22 benzene ring.

*D*—H⋯*A*	*D*—H	H⋯*A*	*D*⋯*A*	*D*—H⋯*A*
O3—H1⋯O2	0.96	1.62	2.5121 (11)	152
C5—H5*A*⋯O3^i^	0.93	2.47	3.3912 (13)	170
C18—H18*A*⋯O3^ii^	0.93	2.48	3.1235 (16)	127
C7—H7*B*⋯*Cg*1^i^	0.97	2.75	3.6633 (11)	158

Symmetry codes: (i) 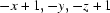; (ii) 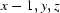.

**Fig. 1.**
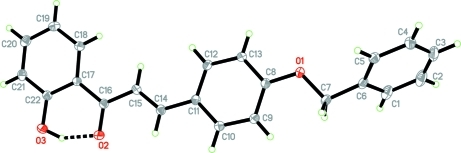
The molecular structure of the title compound, showing 50% probability displacement ellipsoids and the atom-numbering scheme. The dashed line indicates the intramolecular hydrogen bond.

**Fig. 2.**
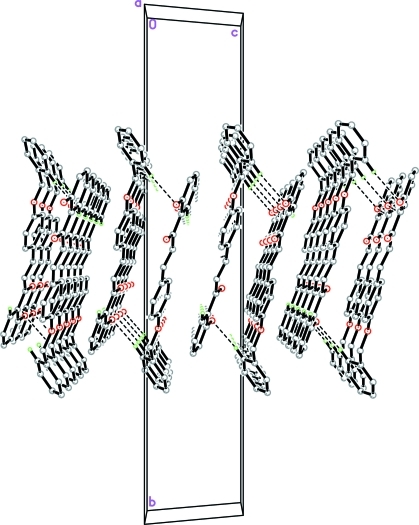
The crystal packing of the title compound, viewed along the a axis. H atoms not involved in the intermolecular interactions (dashed lines) have been omitted for clarity.

## supplementary crystallographic information

### Comment

Chalcones (1,3-diarylpropenones) have been widely used as starting materials in numerous synthetic reactions (Awad *et al.*, 1960; Coudert *et al.*, 1988) including the preparation of fused ring heterocyclic compounds (Insuasty *et al.*, 1992, 1997; Kolos *et al.*, 1996; Samshuddin *et al.*, 2010; Fun *et al.*, 2010). Chalcones are also finding application as organic nonlinear optical materials (NLO) for their SHG conversion efficiency (Sarojini *et al.*, 2006; Shettigar *et al.*, 2010). The crystal structures of some of the related chalcones *viz*: 1-(3,4-dimethoxyphenyl)-3-(3-methylphenyl)prop-2-en-1-one (Sharma *et al.*, 1997), 3-(3,4-dimethoxyphenyl)-1-(4-hydroxy-phenyl)prop-2-en-1-one (Ravishankar *et al.*, 2003), 1-(4-chlorophenyl)-3-(4-hydroxyphenyl)prop-2-en-1-one (Ravishankar *et al.*, 2005), 3-(3,4-dimethoxyphenyl)-1-(4-fluorophenyl)prop-2-en-1-one (Butcher *et al.*, 2006), 3-(2-chlorophenyl)-1-(4-hydroxyphenyl)prop-2-en-1-one (Narayana *et al.*, 2007), (2*E*)-1-(2-hydroxyphenyl)-3-(4-methoxy-phenyl)prop-2-en-1-one, (2*E*)-1-(2-hydroxyphenyl)-3-[4-(methyl-sulfanyl)phenyl]prop-2-en-1-one (Sarojini *et al.*, 2007**a*,b*) and (2*E*)-3-(2-anthryl)-1-(2-hydroxyphenyl)prop-2-en-1-one (Jasinski *et al.*, 2011)  have been reported. In a continuation of our studies of the structures of chalcones, we report the crystal structure of a new chalcone, (2*E*)-3-(3-benzyloxyphenyl)-1-(2-hydroxyphenyl)prop-2-en-1-one, C_22_H_18_O_3_, (I).

The molecular strucure of (I) is shown in Fig. 1. An intramolecular O3—H1···O2 hydrogen bond (Table 1) stabilized the molecular structure, forming an *S*(6) ring motif (Bernstein *et al.*, 1995). The (C8–C13) benzene ring forms a dihedral angle of 64.74 (5)° with the C1–C6 phenyl ring and is almost co-planar with the C17–C22 phenyl ring with a dihedral angle of 5.58 (5)°. Bond lengths (Allen *et al.*, 1987) and angles are within the normal ranges.

In the crystal packing (Fig. 2), the molecules are linked into columns along the *a* axis *via* intermolecular C5—H5A···O3 and C18—H18A···O3 hydrogen bonds (Table 1). C–H···π interactions (Table 1) involving the centroids of C17–C22 rings (*Cg*1) further stabilize the crystal structure.

### Experimental

2-Hydroxyacetophenone (1.36 g, 0.01 mol) was mixed with 4-benzyloxybenzaldehyde (2.12 g, 0.01 mol) and dissolved in ethanol (40 ml). To this solution 4 ml of KOH (50%) was added at 278 K. The reaction mixture was stirred for 8 h and poured onto crushed ice. The pH of this mixture was adjusted to 3–4 with 2 *M* HCl aqueous solution. The resulting crude yellow solid was filtered, washed successively with dilute HCl solution and distilled water and finally recrystallized from ethanol (95%) to give the pure chalcone. Crystals suitable for X-ray diffraction studies were grown by the slow evaporation of the solution of the compound in ethyl alcohol (*m. p.*: 367–369 K). Composition: Found (calculated) for C_22_H_18_O_3_, C 79.98 (79.93); H: 5.49 (5.52).

### Refinement

H1 was located from the difference Fourier map and was fixed at its found position with *U*_iso_(H) = 1.5 *U*_eq_(O) [O–H = 0.9618 Å]. The remaining H atoms were positioned geometrically and refined using a riding model with *U*_iso_(H) = 1.2 *U*_eq_(C) [C–H = 0.93–0.97 Å].

### Crystal data

**Table:**

C_22_H_18_O_3_	*F*(000) = 696
*M**_r_* = 330.36	*D*_x_ = 1.335 Mg m^−^^3^
Monoclinic, *P*2_1_/*c*	Mo *K*α radiation, λ = 0.71073 Å
Hall symbol: -P 2ybc	Cell parameters from 9442 reflections
*a* = 6.6343 (1) Å	θ = 3.0–32.7°
*b* = 35.7706 (5) Å	µ = 0.09 mm^−^^1^
*c* = 8.1537 (1) Å	*T* = 100 K
β = 121.879 (1)°	Block, yellow
*V* = 1643.12 (4) Å^3^	0.46 × 0.41 × 0.28 mm
*Z* = 4	

### Data collection

**Table:**

Bruker SMART APEXII CCD area-detector diffractometer	5994 independent reflections
Radiation source: fine-focus sealed tube	5154 reflections with *I* > 2σ(*I*)
graphite	*R*_int_ = 0.024
φ and ω scans	θ_max_ = 32.7°, θ_min_ = 2.3°
Absorption correction: multi-scan (*SADABS*; Bruker, 2009)	*h* = −10→9
*T*_min_ = 0.960, *T*_max_ = 0.976	*k* = −54→54
23662 measured reflections	*l* = −12→12

### Refinement

**Table:**

Refinement on *F*^2^	Primary atom site location: structure-invariant direct methods
Least-squares matrix: full	Secondary atom site location: difference Fourier map
*R*[*F*^2^ > 2σ(*F*^2^)] = 0.048	Hydrogen site location: inferred from neighbouring sites
*wR*(*F*^2^) = 0.126	H-atom parameters constrained
*S* = 1.04	*w* = 1/[σ^2^(*F*_o_^2^) + (0.0608*P*)^2^ + 0.6186*P*] where *P* = (*F*_o_^2^ + 2*F*_c_^2^)/3
5994 reflections	(Δ/σ)_max_ < 0.001
226 parameters	Δρ_max_ = 0.47 e Å^−^^3^
0 restraints	Δρ_min_ = −0.21 e Å^−^^3^

### Special details

**Table:**

Experimental. The crystal was placed in the cold stream of an Oxford Cryosystems Cobra open-flow nitrogen cryostat (Cosier & Glazer, 1986) operating at 100.0 (1) K.
Geometry. All e.s.d.'s (except the e.s.d. in the dihedral angle between two l.s. planes) are estimated using the full covariance matrix. The cell e.s.d.'s are taken into account individually in the estimation of e.s.d.'s in distances, angles and torsion angles; correlations between e.s.d.'s in cell parameters are only used when they are defined by crystal symmetry. An approximate (isotropic) treatment of cell e.s.d.'s is used for estimating e.s.d.'s involving l.s. planes.
Refinement. Refinement of *F*^2^ against ALL reflections. The weighted *R*-factor *wR* and goodness of fit *S* are based on *F*^2^, conventional *R*-factors *R* are based on *F*, with *F* set to zero for negative *F*^2^. The threshold expression of *F*^2^ > σ(*F*^2^) is used only for calculating *R*-factors(gt) *etc*. and is not relevant to the choice of reflections for refinement. *R*-factors based on *F*^2^ are statistically about twice as large as those based on *F*, and *R*- factors based on ALL data will be even larger.

### Fractional atomic coordinates and isotropic or equivalent isotropic displacement parameters (Å^2^)

**Table:**

	*x*	*y*	*z*	*U*_iso_*/*U*_eq_	
O1	0.22678 (13)	−0.12255 (2)	0.64011 (11)	0.01788 (14)	
O2	1.16788 (13)	0.04531 (2)	0.73546 (11)	0.01926 (15)	
O3	1.37772 (13)	0.10727 (2)	0.82954 (12)	0.01960 (15)	
H1	1.3290	0.0824	0.7790	0.029*	
C1	0.1804 (2)	−0.21718 (3)	0.69912 (16)	0.0233 (2)	
H1A	0.3438	−0.2209	0.7668	0.028*	
C2	0.0347 (3)	−0.24325 (3)	0.71398 (18)	0.0297 (3)	
H2A	0.1013	−0.2641	0.7926	0.036*	
C3	−0.2084 (2)	−0.23818 (3)	0.61239 (18)	0.0283 (2)	
H3A	−0.3055	−0.2557	0.6219	0.034*	
C4	−0.3076 (2)	−0.20672 (3)	0.49557 (17)	0.0238 (2)	
H4A	−0.4713	−0.2033	0.4264	0.029*	
C5	−0.16259 (19)	−0.18037 (3)	0.48208 (15)	0.01870 (18)	
H5A	−0.2296	−0.1593	0.4053	0.022*	
C6	0.08311 (18)	−0.18549 (3)	0.58347 (14)	0.01668 (17)	
C7	0.24046 (18)	−0.15803 (3)	0.56323 (14)	0.01653 (17)	
H7A	0.4030	−0.1670	0.6331	0.020*	
H7B	0.1891	−0.1553	0.4282	0.020*	
C8	0.37550 (16)	−0.09508 (3)	0.64855 (13)	0.01453 (16)	
C9	0.53008 (17)	−0.09903 (3)	0.58253 (14)	0.01643 (17)	
H9A	0.5357	−0.1213	0.5262	0.020*	
C10	0.67596 (17)	−0.06915 (3)	0.60209 (14)	0.01585 (17)	
H10A	0.7794	−0.0719	0.5585	0.019*	
C11	0.67167 (16)	−0.03522 (3)	0.68526 (13)	0.01424 (16)	
C12	0.51140 (17)	−0.03170 (3)	0.74868 (14)	0.01541 (17)	
H12A	0.5032	−0.0093	0.8028	0.018*	
C13	0.36650 (17)	−0.06120 (3)	0.73120 (14)	0.01546 (17)	
H13A	0.2626	−0.0586	0.7744	0.019*	
C14	0.83060 (17)	−0.00553 (3)	0.70116 (13)	0.01512 (17)	
H14A	0.9183	−0.0100	0.6443	0.018*	
C15	0.86482 (17)	0.02778 (3)	0.78915 (14)	0.01564 (17)	
H15A	0.7793	0.0338	0.8463	0.019*	
C16	1.03580 (16)	0.05459 (3)	0.79541 (13)	0.01431 (16)	
C17	1.05437 (16)	0.09259 (3)	0.87343 (13)	0.01351 (16)	
C18	0.90277 (17)	0.10566 (3)	0.93231 (14)	0.01700 (17)	
H18A	0.7872	0.0898	0.9246	0.020*	
C19	0.92093 (19)	0.14160 (3)	1.00144 (15)	0.02028 (19)	
H19A	0.8178	0.1497	1.0388	0.024*	
C20	1.09498 (19)	0.16556 (3)	1.01480 (15)	0.01988 (19)	
H20A	1.1082	0.1897	1.0618	0.024*	
C21	1.24825 (18)	0.15361 (3)	0.95848 (14)	0.01802 (18)	
H21A	1.3642	0.1697	0.9682	0.022*	
C22	1.22862 (16)	0.11736 (3)	0.88693 (13)	0.01474 (17)	

### Atomic displacement parameters (Å^2^)

**Table:**

	*U*^11^	*U*^22^	*U*^33^	*U*^12^	*U*^13^	*U*^23^
O1	0.0210 (3)	0.0121 (3)	0.0250 (4)	−0.0031 (2)	0.0152 (3)	−0.0037 (3)
O2	0.0199 (3)	0.0171 (3)	0.0267 (4)	−0.0001 (3)	0.0163 (3)	−0.0017 (3)
O3	0.0174 (3)	0.0188 (3)	0.0277 (4)	−0.0011 (3)	0.0154 (3)	−0.0008 (3)
C1	0.0310 (5)	0.0153 (4)	0.0192 (5)	0.0003 (4)	0.0103 (4)	0.0007 (3)
C2	0.0488 (7)	0.0159 (5)	0.0248 (5)	−0.0033 (5)	0.0197 (5)	0.0015 (4)
C3	0.0467 (7)	0.0181 (5)	0.0315 (6)	−0.0120 (5)	0.0284 (5)	−0.0067 (4)
C4	0.0291 (5)	0.0209 (5)	0.0293 (5)	−0.0068 (4)	0.0207 (5)	−0.0066 (4)
C5	0.0239 (5)	0.0151 (4)	0.0212 (4)	−0.0005 (3)	0.0148 (4)	−0.0015 (3)
C6	0.0233 (4)	0.0123 (4)	0.0156 (4)	−0.0013 (3)	0.0110 (3)	−0.0024 (3)
C7	0.0195 (4)	0.0131 (4)	0.0177 (4)	0.0000 (3)	0.0103 (3)	−0.0017 (3)
C8	0.0145 (4)	0.0131 (4)	0.0155 (4)	−0.0004 (3)	0.0076 (3)	−0.0002 (3)
C9	0.0179 (4)	0.0145 (4)	0.0181 (4)	−0.0010 (3)	0.0104 (3)	−0.0028 (3)
C10	0.0167 (4)	0.0157 (4)	0.0165 (4)	−0.0002 (3)	0.0098 (3)	−0.0009 (3)
C11	0.0145 (4)	0.0133 (4)	0.0140 (4)	0.0004 (3)	0.0069 (3)	0.0009 (3)
C12	0.0168 (4)	0.0121 (4)	0.0171 (4)	0.0007 (3)	0.0089 (3)	0.0001 (3)
C13	0.0167 (4)	0.0139 (4)	0.0179 (4)	0.0010 (3)	0.0105 (3)	−0.0001 (3)
C14	0.0147 (4)	0.0146 (4)	0.0153 (4)	0.0006 (3)	0.0074 (3)	0.0018 (3)
C15	0.0163 (4)	0.0153 (4)	0.0169 (4)	−0.0005 (3)	0.0098 (3)	0.0005 (3)
C16	0.0140 (4)	0.0141 (4)	0.0146 (4)	0.0005 (3)	0.0074 (3)	0.0010 (3)
C17	0.0129 (4)	0.0134 (4)	0.0146 (4)	0.0005 (3)	0.0075 (3)	0.0003 (3)
C18	0.0166 (4)	0.0177 (4)	0.0193 (4)	0.0002 (3)	0.0113 (3)	−0.0008 (3)
C19	0.0217 (5)	0.0207 (5)	0.0215 (5)	0.0022 (4)	0.0136 (4)	−0.0025 (3)
C20	0.0237 (5)	0.0169 (4)	0.0169 (4)	0.0007 (3)	0.0093 (4)	−0.0022 (3)
C21	0.0184 (4)	0.0157 (4)	0.0178 (4)	−0.0034 (3)	0.0080 (3)	−0.0017 (3)
C22	0.0130 (4)	0.0162 (4)	0.0146 (4)	0.0004 (3)	0.0070 (3)	0.0011 (3)

### Geometric parameters (Å, °)

**Table:**

O1—C8	1.3683 (11)	C10—C11	1.3978 (13)
O1—C7	1.4395 (12)	C10—H10A	0.9300
O2—C16	1.2528 (11)	C11—C12	1.4123 (13)
O3—C22	1.3477 (11)	C11—C14	1.4535 (13)
O3—H1	0.9618	C12—C13	1.3833 (13)
C1—C2	1.3942 (17)	C12—H12A	0.9300
C1—C6	1.3956 (14)	C13—H13A	0.9300
C1—H1A	0.9300	C14—C15	1.3467 (13)
C2—C3	1.382 (2)	C14—H14A	0.9300
C2—H2A	0.9300	C15—C16	1.4656 (13)
C3—C4	1.3946 (17)	C15—H15A	0.9300
C3—H3A	0.9300	C16—C17	1.4780 (13)
C4—C5	1.3924 (14)	C17—C18	1.4038 (13)
C4—H4A	0.9300	C17—C22	1.4139 (13)
C5—C6	1.3966 (15)	C18—C19	1.3833 (14)
C5—H5A	0.9300	C18—H18A	0.9300
C6—C7	1.5044 (14)	C19—C20	1.3952 (15)
C7—H7A	0.9700	C19—H19A	0.9300
C7—H7B	0.9700	C20—C21	1.3860 (15)
C8—C9	1.3945 (13)	C20—H20A	0.9300
C8—C13	1.4031 (13)	C21—C22	1.3996 (14)
C9—C10	1.3933 (13)	C21—H21A	0.9300
C9—H9A	0.9300		
			
C8—O1—C7	116.46 (7)	C10—C11—C14	118.56 (8)
C22—O3—H1	104.7	C12—C11—C14	123.57 (8)
C2—C1—C6	120.49 (11)	C13—C12—C11	120.71 (9)
C2—C1—H1A	119.8	C13—C12—H12A	119.6
C6—C1—H1A	119.8	C11—C12—H12A	119.6
C3—C2—C1	120.24 (11)	C12—C13—C8	120.33 (9)
C3—C2—H2A	119.9	C12—C13—H13A	119.8
C1—C2—H2A	119.9	C8—C13—H13A	119.8
C2—C3—C4	119.74 (11)	C15—C14—C11	127.18 (9)
C2—C3—H3A	120.1	C15—C14—H14A	116.4
C4—C3—H3A	120.1	C11—C14—H14A	116.4
C5—C4—C3	120.24 (11)	C14—C15—C16	120.47 (9)
C5—C4—H4A	119.9	C14—C15—H15A	119.8
C3—C4—H4A	119.9	C16—C15—H15A	119.8
C4—C5—C6	120.25 (10)	O2—C16—C15	120.07 (9)
C4—C5—H5A	119.9	O2—C16—C17	119.68 (8)
C6—C5—H5A	119.9	C15—C16—C17	120.26 (8)
C1—C6—C5	119.03 (10)	C18—C17—C22	117.95 (9)
C1—C6—C7	120.27 (9)	C18—C17—C16	122.79 (8)
C5—C6—C7	120.66 (9)	C22—C17—C16	119.25 (8)
O1—C7—C6	108.71 (8)	C19—C18—C17	121.67 (9)
O1—C7—H7A	109.9	C19—C18—H18A	119.2
C6—C7—H7A	109.9	C17—C18—H18A	119.2
O1—C7—H7B	109.9	C18—C19—C20	119.54 (9)
C6—C7—H7B	109.9	C18—C19—H19A	120.2
H7A—C7—H7B	108.3	C20—C19—H19A	120.2
O1—C8—C9	124.44 (8)	C21—C20—C19	120.43 (9)
O1—C8—C13	115.60 (8)	C21—C20—H20A	119.8
C9—C8—C13	119.96 (9)	C19—C20—H20A	119.8
C10—C9—C8	119.09 (9)	C20—C21—C22	120.05 (9)
C10—C9—H9A	120.5	C20—C21—H21A	120.0
C8—C9—H9A	120.5	C22—C21—H21A	120.0
C9—C10—C11	122.04 (9)	O3—C22—C21	117.97 (9)
C9—C10—H10A	119.0	O3—C22—C17	121.68 (9)
C11—C10—H10A	119.0	C21—C22—C17	120.35 (9)
C10—C11—C12	117.87 (8)		
			
C6—C1—C2—C3	0.73 (17)	C9—C8—C13—C12	−0.39 (14)
C1—C2—C3—C4	−0.38 (17)	C10—C11—C14—C15	−174.62 (9)
C2—C3—C4—C5	−0.40 (17)	C12—C11—C14—C15	5.66 (15)
C3—C4—C5—C6	0.83 (16)	C11—C14—C15—C16	178.92 (9)
C2—C1—C6—C5	−0.30 (15)	C14—C15—C16—O2	−6.92 (14)
C2—C1—C6—C7	−178.15 (10)	C14—C15—C16—C17	173.44 (9)
C4—C5—C6—C1	−0.47 (15)	O2—C16—C17—C18	176.07 (9)
C4—C5—C6—C7	177.37 (9)	C15—C16—C17—C18	−4.29 (14)
C8—O1—C7—C6	174.51 (8)	O2—C16—C17—C22	−2.76 (13)
C1—C6—C7—O1	−117.15 (10)	C15—C16—C17—C22	176.88 (8)
C5—C6—C7—O1	65.04 (11)	C22—C17—C18—C19	0.09 (14)
C7—O1—C8—C9	2.20 (13)	C16—C17—C18—C19	−178.75 (9)
C7—O1—C8—C13	−177.49 (8)	C17—C18—C19—C20	−0.48 (15)
O1—C8—C9—C10	−178.92 (9)	C18—C19—C20—C21	0.32 (16)
C13—C8—C9—C10	0.75 (14)	C19—C20—C21—C22	0.24 (15)
C8—C9—C10—C11	−0.25 (15)	C20—C21—C22—O3	178.42 (9)
C9—C10—C11—C12	−0.60 (14)	C20—C21—C22—C17	−0.64 (14)
C9—C10—C11—C14	179.67 (9)	C18—C17—C22—O3	−178.55 (9)
C10—C11—C12—C13	0.97 (14)	C16—C17—C22—O3	0.33 (14)
C14—C11—C12—C13	−179.32 (9)	C18—C17—C22—C21	0.47 (14)
C11—C12—C13—C8	−0.49 (14)	C16—C17—C22—C21	179.36 (8)
O1—C8—C13—C12	179.31 (8)		

### Hydrogen-bond geometry (Å, °)

**Table:**

Cg is the centroid of the C17–C22 benzene ring.

**Table:**

*D*—H···*A*	*D*—H	H···*A*	*D*···*A*	*D*—H···*A*
O3—H1···O2	0.96	1.62	2.5121 (11)	152
C5—H5A···O3^i^	0.93	2.47	3.3912 (13)	170
C18—H18A···O3^ii^	0.93	2.48	3.1235 (16)	127
C7—H7B···Cg1^i^	0.97	2.75	3.6633 (11)	158

Symmetry codes: (i) −*x*+1, −*y*, −*z*+1; (ii) *x*−1, *y*, *z*.
